# Supplementary material for: PRL2 regulates neutrophil extracellular trap formation which contributes to severe malaria and acute lung injury
Source: Nat Commun. 2024 Jan 29;15:881. doi: 10.1038/s41467-024-45210-5 (PMC10825202; doi:10.1038/s41467-024-45210-5)

# PRL2 regulates neutrophil extracellular trap formation which contributes to severe malaria and acute lung injury

Supplementary information

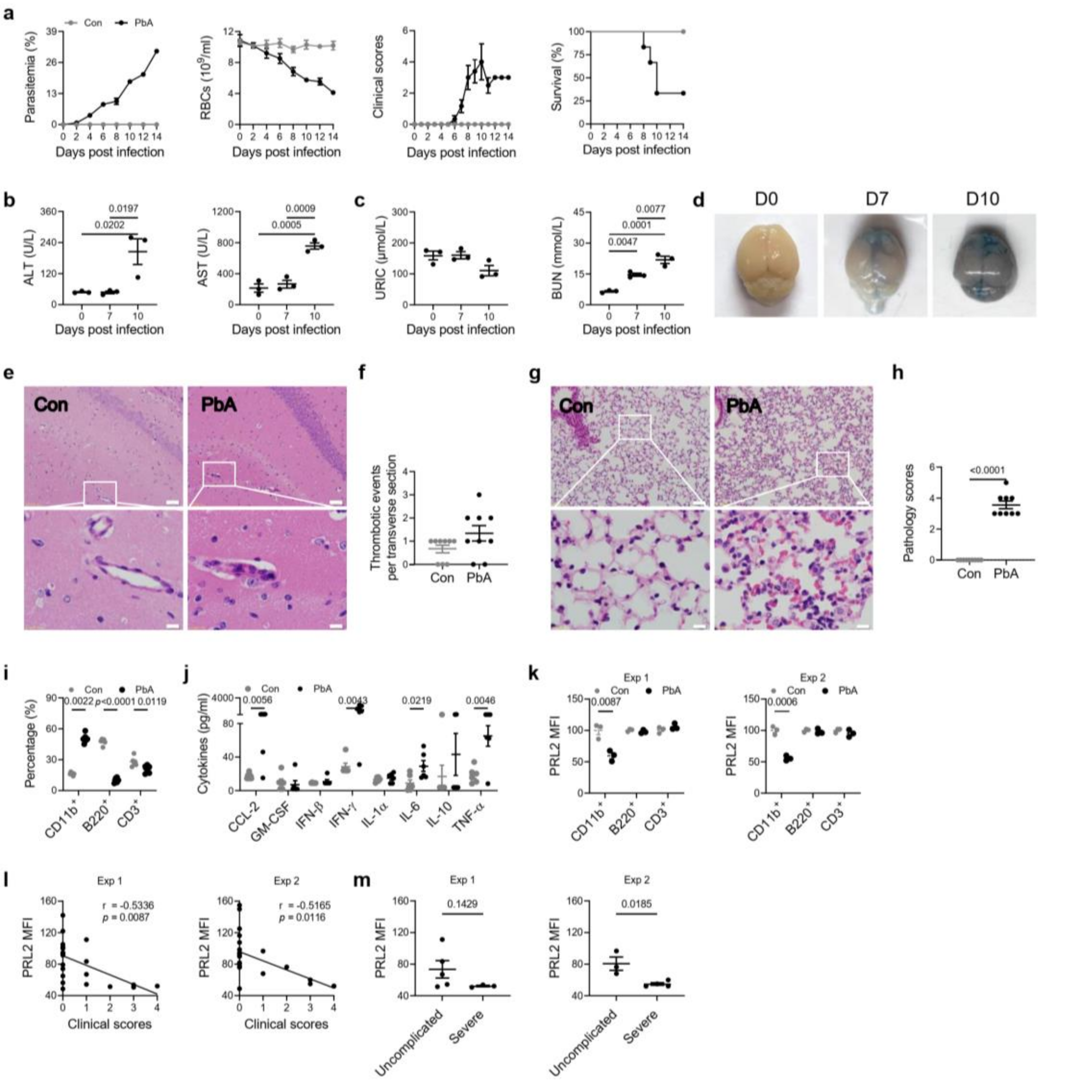


**Supplementary Fig. 1 Severe malaria occurs during *Plasmodium berghei* ANKA infection, related to Figure 1.**

C57BL/6J mice were infected with $1\times10^6$ *P. berghei* ANKA (PbA) iRBCs. **a** Parasitemia, number of RBCs, clinical scores and survival curve of normal control (Con) and *P. berghei* ANKA (PbA) infected mice during the 14-day infection (n = 6 mice per group). Data were pooled from two independent experiments. Serum levels of **b** alanine aminotransferase (ALT), aspartate aminotransferase (AST), **c** uric acid (URIC) and blood urea nitrogen (BUN) from PbA infected mice at the indicated times (n = 3 mice per time point). **d** PbA infected mice were injected with 2% Evans blue dye 1 h prior to dissection at the indicated times. Representative images of brains are shown (n = 3 mice per time point). **e-h** Brain and lung tissues stained with hematoxylin-eosin (H&E) from normal control and PbA infected mice at 7 days post infection (dpi) (n = 9 mice per

group). Data were pooled from three independent experiments. **e** Representative images of brain tissues. Scale bars, up: 50 µm, down: 10 µm. **f** Quantification of cerebral thrombotic areas. **g** Representative images of lung tissues. Scale bars, up: 50 µm, down: 10 µm. **h** Quantification of pulmonary pathology scores. **i-k** Normal control and PbA infected mice at 7 dpi (n = 6 mice per group). Data were pooled from two independent experiments. **i** Percentage of different subsets of peripheral blood cells. **j** Concentrations of serum cytokines and chemokines. **k** Relative PRL2 mean fluorescence intensity (MFI) in different subsets of peripheral blood cells, normalized to normal mice. **l** Spearman's correlation analysis between PRL2 MFI in $CD11b^+$ peripheral blood cells and the clinical scores of PbA infected mice (n = 46, samples from 10 mice at 0, 2, 4, 6, 8 dpi). **m** Relative PRL2 MFI in $CD11b^+$ peripheral blood cells from mice with uncomplicated and severe malaria, samples as in **l**. **k-m** Data show the separate results of two independent experiments. Pooled data are shown in main Figure **1a-c**. Data are presented as the mean ± SEM. *p* values were calculated by one-way ANOVA with Tukey's multiple testing (**b**, **c**), two-tailed Mann-Whitney test (**h**), two-tailed unpaired t test (**i**, **j**, **k**, **m**) or Spearman's correlation (**l**) and shown in the figures. Source data are provided as a Source Data file.

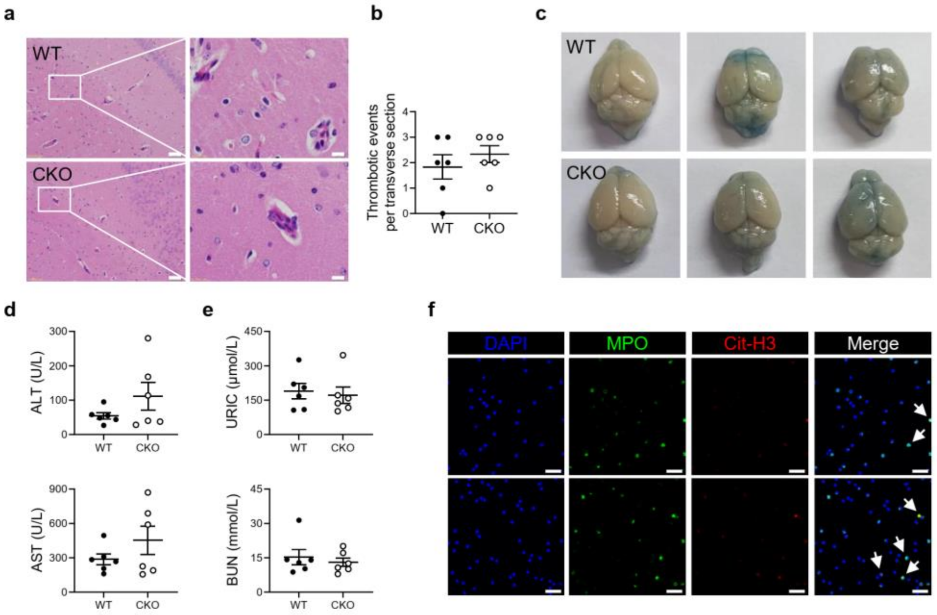


**Supplementary Fig. 2 Similar brain, liver and kidney injury between WT and PRL2 myeloid CKO mice after PbA infection, related to Figure 1.**

Wildtype (WT) and PRL2 myeloid cell conditional knockout (CKO) mice were infected with $1\times10^6$ *P. berghei* ANKA (PbA) iRBCs. **a** Representative images of brain tissues stained with hematoxylin-eosin (H&E) from PbA infected WT and PRL2 myeloid CKO mice at 7 days post infection (dpi) (n = 6 mice per group). Scale bars, left: 50 μm, right: 10 μm. **b** Quantification of cerebral thrombotic areas from **a**. **c** PbA infected WT and PRL2 CKO mice were injected with 2% Evans blue dye 1 h prior to dissection at 7 dpi (n = 6 mice per group). Representative images of brains are shown. Serum levels of **d** alanine aminotransferase (ALT), aspartate aminotransferase (AST), **e** uric acid (URIC) and blood urea nitrogen (BUN) from PbA infected WT and PRL2 myeloid CKO mice at 7 dpi (n = 6 mice per group). **f** Representative immunofluorescence images of peripheral blood cells from PbA infected WT and PRL2 myeloid CKO mice at 7 dpi (n = 6 mice per group). DNA is stained in blue (DAPI), myeloperoxidase is stained in green (MPO) and citrullinated histone H3 is stained in red (Cit-H3). Scale bars, 20 μm. Neutrophils are indicated as co-stained with MPO and DAPI. Neutrophil extracellular traps (NETs) are indicated as co-stained with MPO and Cit-H3. All data were pooled from two independent experiments. Data are presented as the mean ± SEM. Source data are provided as a Source Data file.

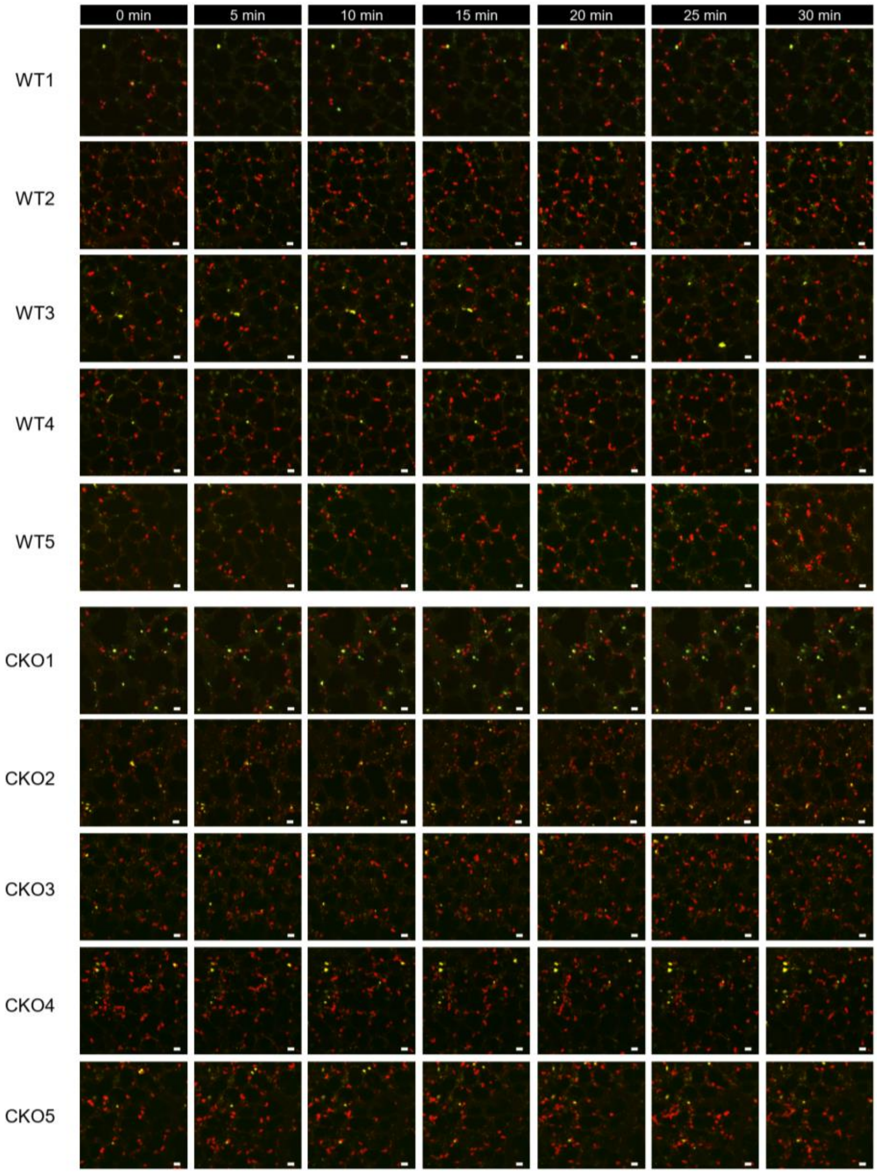


**Supplementary Fig. 3 PRL2 myeloid CKO mice showed more severe malaria related lung injury with increased NET formation via intravital multiphoton microscopy, related to Figure 2.**

Wildtype (WT) and PRL2 myeloid cell conditional knockout (CKO) mice were infected with $1\times10^6$ *P. berghei* ANKA (PbA) iRBCs. Intravital imaging of the lungs at 7 dpi. Time-lapse images are shown (n = 6 mice per group, in addition to **Fig.2c**, another 5 mice are shown). Scale bars, 20 μm. Neutrophils are stained in red (Ly6G) and extracellular DNA is stained in green (Sytox Green, SG). Source data are provided as a Source Data file.

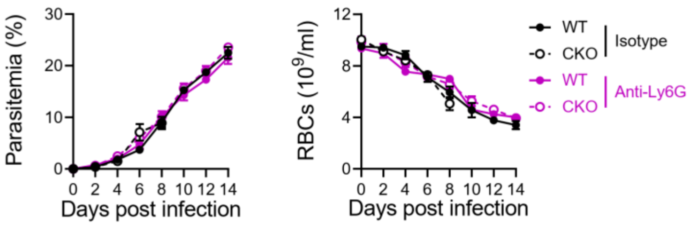


**Supplementary Fig. 4 Depletion of neutrophils does not affect the parasitemia and number of RBCs after PbA infection, related to Figure 3.**

*P. berghei* ANKA (PbA) infected wildtype (WT) and PRL2 myeloid cell conditional knockout (CKO) mice were intraperitoneally injected with anti-Ly6G monoclonal antibody or an isotype control at 6 days post infection (dpi). Parasitemia and RBCs number of the mice described above during the 14-day infection (n = 8 mice per group). Data were pooled from two independent experiments. Data are presented as the mean ± SEM. Source data are provided as a Source Data file.

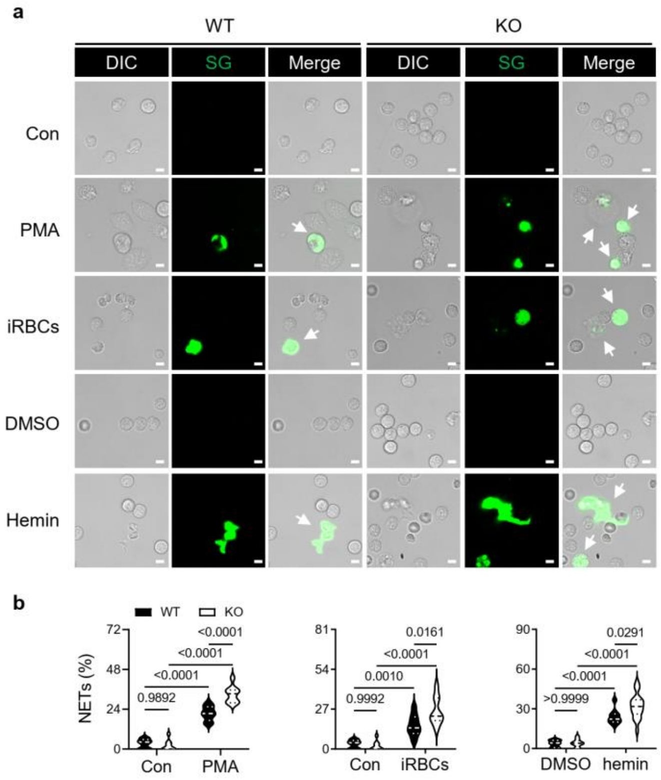


**Supplementary Fig. 5 Neutrophils with PRL2 deficiency have enhanced ability to form NET, related to Figure 4.**

**a** Representative immunofluorescence images of neutrophil extracellular traps (NETs) released by wildtype (WT) and PRL2 knockout (KO) bone marrow derived neutrophils (BMNs) after PMA, iRBC and hemin stimulation. Living cells were stained with Sytox Green (SG) to show extracellular DNA. Scale bar, 5 µm. DIC, differential interference contrast. Arrowheads indicate NETs. **b** Quantification of NETs in **a** is shown. Data were pooled from three independent experiments. BMNs were collected from different mice (n = 3 per group). Violin plots show the median and interquartile range. *p* values were calculated by two-way ANOVA with Tukey's multiple testing (**b**) and shown in the figures. Source data are provided as a Source Data file.

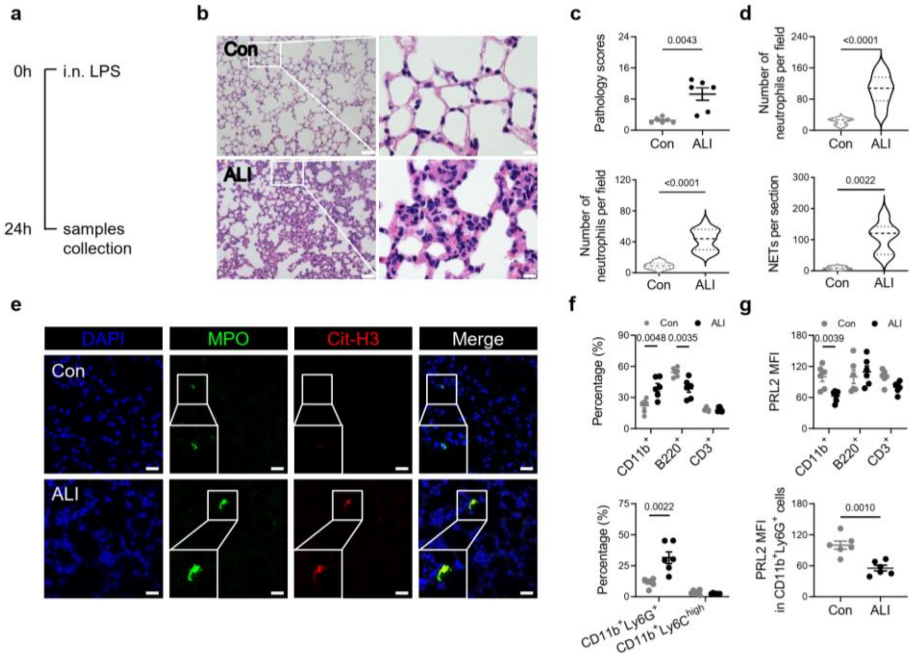


**Supplementary Fig. 6 PRL2 related NET formation contributes to the pathology in LPS induced ALI, related to Figure 6.**

**a** A schematic showing the experimental design for LPS induced acute lung injury (ALI) model. **b-g** C57BL/6J mice were induced ALI in **a** or grouped as normal control (Con) (n = 6 mice per group). **b** Representative images of lung tissues stained with hematoxylin-eosin (H&E). Scale bars, left: 50 μm, right: 10 μm. **c** Pulmonary pathology scores and infiltrated neutrophil numbers were quantified from **b**. **d** Quantification of neutrophils and neutrophil extracellular traps (NETs) from immunofluorescence images of lungs. **e** Representative immunofluorescence images of lungs as described in **d**. DNA is stained in blue (DAPI), myeloperoxidase is stained in green (MPO) and citrullinated histone H3 is stained in red (Cit-H3). Scale bars, 20 μm. Neutrophils are indicated as co-stained with MPO and DAPI. NETs are indicated as co-stained with MPO and Cit-H3. **f** Percentage of different subsets of peripheral blood cells. **g** Relative PRL2 mean fluorescence intensity (MFI) in different subsets of peripheral blood cells, normalized to normal mice. All data were pooled from two independent experiments. Data are presented as the mean ± SEM or in violin plots showing the median and interquartile range. *p* values were calculated by two-tailed Mann-Whitney test (**c**, **d**, **f** down) or two-tailed unpaired t test (**f** up, **g**) and shown in the figures. Source Data are provided as a Source Data file.

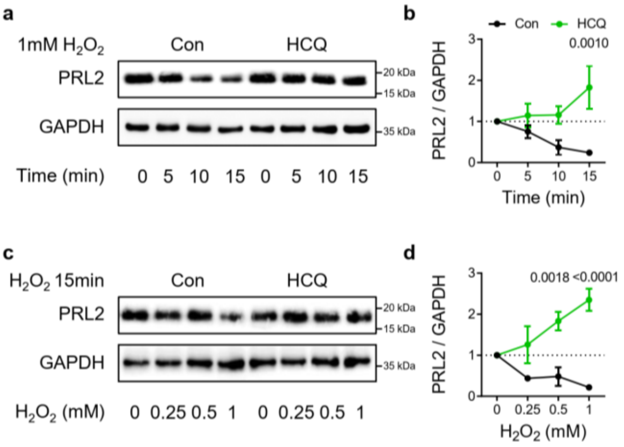


**Supplementary Fig. 7 HCQ blocks PRL2 degradation *in vitro*, related to Figure 7.** Mice bone marrow derived neutrophils (BMNs) were isolated and pre-treated with 50 μM hydroxychloroquine (HCQ) or PBS for 1 h. **a** Representative immunoblot images and **b** quantification of PRL2 protein levels in BMNs treated with 1 mM hydrogen peroxide ($H_2O_2$) for the indicated time. **c** Representative immunoblot images and **d** quantification of PRL2 protein levels in BMNs treated with the indicated concentrations of $H_2O_2$ for 15 min. All data were pooled from three independent experiments with similar results. Data are presented as the mean ± SEM. *p* values were calculated by two-way ANOVA with Tukey's multiple testing (**b**, **d**) and shown in the figures. Source data are provided as a Source Data file.

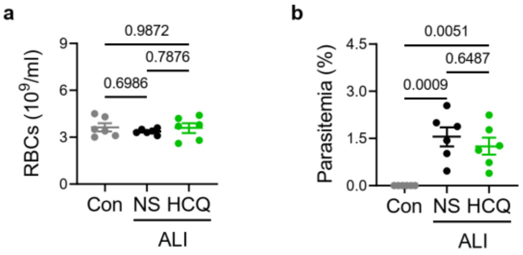


**Supplementary Fig. 8 HCQ administration does not affect peripheral RBCs and parasitemia in iRBCs induced ALI model, related to Figure 7.**

**a** Number of RBCs and **b** parasitemia in peripheral blood from normal control mice, iRBCs induced ALI mice treated with normal saline (NS) or hydroxychloroquine (HCQ) (n = 6 mice per group). Data were pooled from two independent experiments. Data are presented as the mean ± SEM. *p* values were calculated by one-way ANOVA with Tukey's multiple testing (**a**, **b**) and shown in the figures. Source data are provided as a Source Data file.

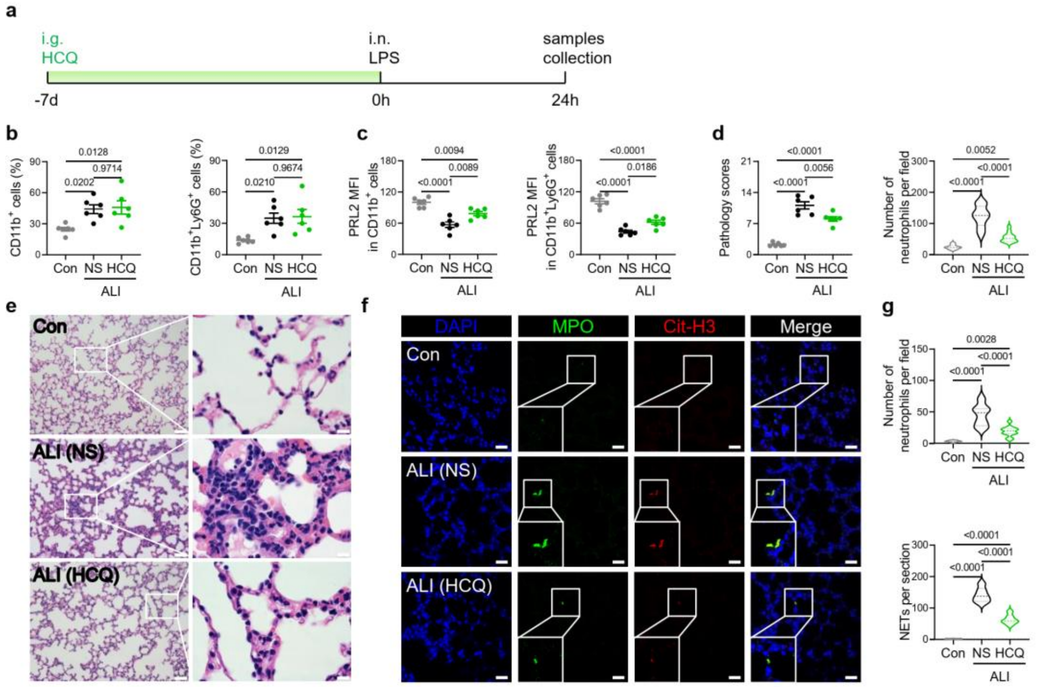


**Supplementary Fig. 9 HCQ alleviated LPS induced lung injury by blocking PRL2 degradation and NET formation, related to Figure 7.**

**a** A flow chart depicting hydroxychloroquine (HCQ) treatment for LPS induced acute lung injury (ALI). **b-g** C57BL/6J mice were treated with normal saline (NS) or HCQ and induced ALI, or just treated with NS and grouped as normal control (Con) (n = 6 mice per group). **b** Percentage of peripheral myeloid cells ($CD11b^{+}$) and neutrophils ($CD11b^{+}Ly6G^{+}$). **c** Relative PRL2 mean fluorescence intensity (MFI) in different subsets of peripheral blood cells, normalized to normal control mice. **d** Pulmonary pathology scores and infiltrated neutrophils numbers were quantified from images of lung tissues stained with hematoxylin-eosin (H&E). **e** Representative histochemical images of lung tissues. Scale bars, left: 50 µm, right: 10 µm. **f** Representative immunofluorescence images of lung tissues. DNA is stained in blue (DAPI), myeloperoxidase is stained in green (MPO) and citrullinated histone H3 is stained in red (Cit-H3). Scale bars, 20 µm. Neutrophils are indicated as co-stained with MPO and DAPI. Neutrophil extracellular traps (NETs) are indicated as co-stained with MPO and Cit-H3. **g** Quantification of neutrophils and NETs from **f**. All data were pooled from two independent experiments. Data are presented as the mean ± SEM or in violin plots showing the median and interquartile range. *p* values were calculated

by one-way ANOVA with Tukey's multiple testing (**b**, **c**, **d**, **g**) and shown in the figures. Source data are provided as a Source Data file.

**Uncropped scans of all blots in Supplementary Figures.**

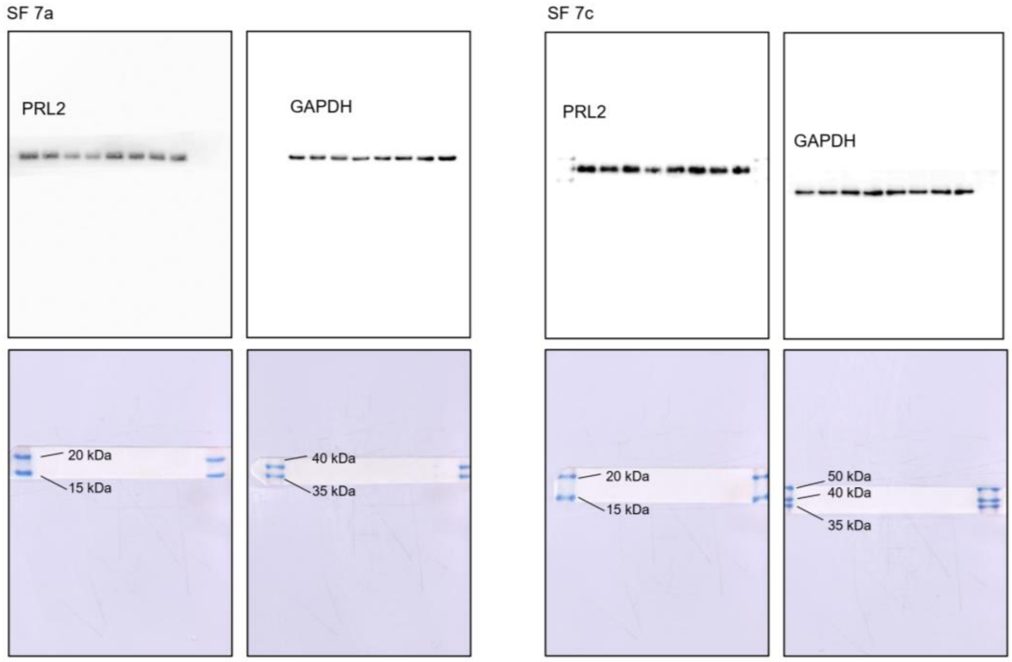

Supplement: Supplementary file 1 — Supplementary Information [file 41467_2024_45210_MOESM1_ESM.pdf]
