# Supplementary material for: PRL2 regulates neutrophil extracellular trap formation which contributes to severe malaria and acute lung injury
Source: Nat Commun. 2024 Jan 29;15:881. doi: 10.1038/s41467-024-45210-5 (PMC10825202; doi:10.1038/s41467-024-45210-5)
Supplement: Supplementary file 3 — Description of Additional Supplementary Files [file 41467_2024_45210_MOESM3_ESM.pdf]

Description of Additional Supplementary Files

File Name: Supplementary Movies 1 & 2

Description: **PRL2 related NET formation is dynamically associated with ALI in malaria, related to Figure 2.** Two-photon imaging of neutrophil extracellular traps formation *in vivo*. Wildtype (WT) and PRL2 myeloid cell conditional knockout (CKO) mice were infected with 1×10^6 *P. berghei* ANKA (PbA) iRBCs. On the 7th dpi, 1 µg rat anti-mouse Ly-6G PE antibody and 5 nmol Sytox Green were delivered via vein injection per mouse. The lungs were imaged every 10 s for 30 min by collecting z-stacks of approximately 6 µm, with a 3 µm step size (n = 6 mice per group from two independent experiments, movies taken in per group, one movie from each of six mice was shown in Supplementary Movie 1, others were shown in Supplementary Movie 2). Scale bar, 20 µm.

File Name: Supplementary Movie 3

Description: **Live cell imaging shows more NETosis in PRL2 KO neutrophils, related to Figure 4.** Representative imaging of neutrophil extracellular traps formation *in vitro*. Wildtype (white) and PRL2 knockout $TdTomato^{+}$ (red) bone marrow neutrophils (BMNs) were mixed equally and stimulated with PMA. Extracellular DNA was stained with Sytox Green (green). Cells were imaged every 1.5 min for 30 min by confocal microscopy. Black/white boxes indicate NETs from WT/KO BMNs, respectively. Scale bar, 25 µm.
